# Design and Evaluation of an Aerosol Electrometer with Low Noise and a Wide Dynamic Range

**DOI:** 10.3390/s18051614

**Published:** 2018-05-18

**Authors:** Yixin Yang, Tongzhu Yu, Jiaoshi Zhang, Wenyu Wang, Huaqiao Gui, Peng Du, Jianguo Liu

**Affiliations:** 1 Key Laboratory of Environmental Optics and Technology, Anhui Institute of Optics and Fine Mechanics, Chinese Academy of Sciences, Hefei 230031, China; yxyang@aiofm.ac.cn (Y.Y.); jszhang@aiofm.ac.cn (J.Z.); wywang@aiofm.ac.cn (W.W.); hqgui@aiofm.ac.cn (H.G.); pdu@aiofm.ac.cn (P.D.); 2 Science Island Branch of Graduate School, University of Science and Technology of China, Hefei 230026, China; 3 CAS Center for Excellence in Regional Atmospheric Environment, Institute of Urban Environment, Chinese Academy of Sciences, Xiamen 361021, China

**Keywords:** aerosol electrometer, wide dynamic range, temperature effect

## Abstract

A low-noise aerosol electrometer with a wide dynamic range has been designed for measuring the total net charge on high concentration aerosol particles within the range of −500 pA to +500 pA. The performance of the aerosol electrometer was evaluated by a series of experiments to determine linearity, sensitivity and noise. The relative errors were controlled within 5.0%, 1.0% and 0.3% at the range of −40 pA to +40 pA, ±40 pA to ±100 pA, and ±100 pA to ±500 pA respectively. The stability of the designed aerosol electrometer was found to be highly sensitive to temperature variations, but under temperature control, the root mean square noise and the peak-to-peak noise were 1.040 fA and 5.2 fA respectively, which are very close to the calculated theoretical limit of the current noise. The excellent correlation and the advantage of a wide dynamic range have been demonstrated by comparing with the designed aerosol electrometer to a commercial aerosol electrometer. A 99.7% (R^2^) statistical correlation was obtained; meanwhile, the designed aerosol electrometer operated well even when an overrange phenomenon appeared in the commercial aerosol electrometer.

## 1. Introduction

Sensors for aerosol measurement make a important role in aerosol science and technology as well as atmospheric pollution monitor. Aerosol electrometers, which are composed of a Faraday cup and an electrometer, have been broadly used in the field of aerosol measurement for decades [1]. One of the primary uses of an aerosol electrometer is to characterize Condensation Particle Counters (CPC) [2,3,4,5,6,7], including characterizing the lower counting limit, the counting efficiency curve and the concentration linearity. Aerosol electrometers have also been used to measure the charge on charged particles as well as determining the size distribution of particles when the aerosol electrometer is combined with a differential mobility analyzer (DMA) [8,9,10,11]. Moreover, aerosol electrometers have been used to measure and sample atmospheric ions and charged aerosols directly [12,13,14], as opposed to other applications that focus more on the total number concentration of particles than on the size distribution [15].

Currently, some applications have been put forward new demands for aerosol electrometer, which require a wide dynamic range. For instance, in-cloud charge measurements require a wide dynamic range, extending from charge in aerosols and dusts to that present in thunderstorms [16]. An electrometer based on the vibrating capacitance of a microelectromechanical systems (MEMS) resonator was used to detect the small currents from ionized particles in an aerosol particle detection system [17]. The main drawback of this application was that the current resolution of the MEMS electrometer was limited by leakage currents, and 1/f noise was as long integration times. Moreover, its dynamic range was narrow and limited to measuring low concentration ionized particles. Two fast electrometers were designed and applied in high-resolution nanoDMA measurements [18]. The response time performance and noise level were both excellent, but still exhibited a limited dynamic range. In that study, the long-term output drift was tested, but the effect of temperature on noise was not discussed. Currently, commercial aerosol electrometers have been developed and broadly applied for years. The TSI Model 3068 Aerosol Electrometer, in particular, is portable and robust for measuring the net charge on aerosol particles in laboratory. It has the advantage of high current accuracy; however, its dynamic range is only −12.5 pA to +12.5 pA [19]. Furthermore, only the main amplifier itself was temperature stabilized by a subminiature proportionally controlled heater. It was also used directly to measure ambient particle charge concentration [12]. The effect of humidity on the zero reading drift of TSI-3068 was tested, but the effect of temperature on the zero reading drift, which is equally important, was not investigated. Intra and Tippayawong [13,14] developed and evaluated a Faraday cup electrometer for measuring and sampling atmospheric ions and charged aerosols. They found the importance of temperature effect and the electrometers were temperature stabilized to about 32 °C. However, they neglect to note that other components in the aerosol electrometer, aside from the electrometer itself, were also affected by temperature variations and generated current noise. Moreover, the sensitivity and noise of the aerosol electrometer were not tested after the temperature was controlled.

The aim of our study was to design a low-noise aerosol electrometer with a wide dynamic range for measuring the total net charge on high concentration aerosol particles. The objectives of this study were to fully discuss the linearity, sensitivity, noise and correlation of the designed aerosol electrometer, and to explore the effect of temperature variations on the noise of the aerosol electrometer.

## 2. Materials and Methods

### 2.1. Description of Aerosol Electrometer 

**Figure 1:**
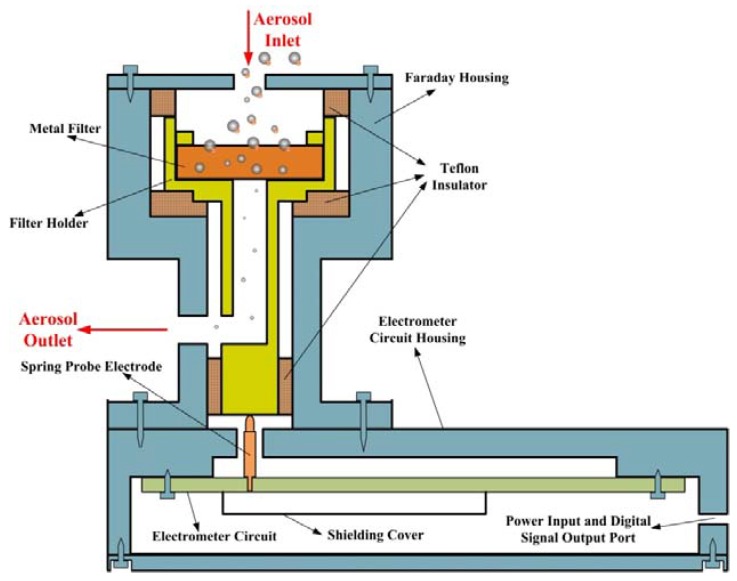
The schematics diagram of the aerosol electrometer.

The schematic diagram of the aerosol electrometer used in this study is shown in Figure 1. This device was composed of an electrometer with a Faraday cup to collect the charges on charged particles. The Faraday cup consisted of a Faraday housing, a metal filter, a metal holder, Teflon insulator, and electrometer circuit housing. To be mentioned, the metal filter used in this paper was sintered copper powder filter element, which was a kind of element made of copper alloy powder sintered at a high temperature. This element has high filtration precision, high mechanical strength, and high utilization of materials. An electrometer circuit with a wide dynamic range and low-noise was also developed to measure ultra-low currents (about 10^−15^ fA). The ultra-low current was produced by the charges on charged particles that were collected by the Faraday cup. In order to reduce the noise in the current measurement, four strategies were employed: (I) A spring probe electrode was used instead of a copper wire to eliminate noise due to vibration. The triboelectric, piezoelectric, and stored charge effects are caused by vibration and the resultant generated current adds to the desired current, causing errors. (II) Electromagnetic interference (EMI) and radio-frequency interference (RFI) from an external source were prevented by incorporating a Faraday housing with a grounded shielding cover made of aluminum. (III) Instead of long-distance wire transmission, the ultra-low current was amplified and converted to digital signal in one, well shielded circuit. (IV) Teflon insulation was used to eliminate noise due to leakage current. The contact surface area between the Teflon insulation and the filter holder should as far as possible small for the sake of alleviating the adverse triboelectric, piezoelectric, and stored charge effects. Additionally, in order to eliminate the loss of charged particles, metal tube fittings (Swagelok) were used in the aerosol inlet and outlet.

**Figure 2:**
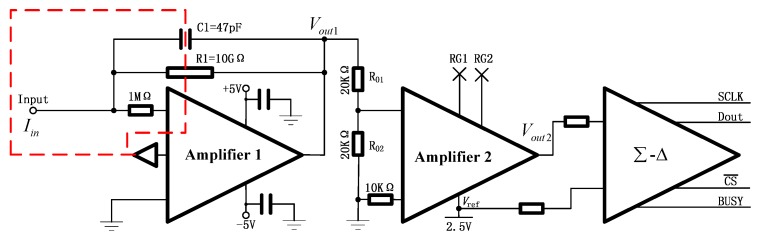
The schematics diagram of electrometer circuit.

The schematic diagram of the electrometer circuit used in this study is shown in Figure 2. First, the input ultra-low current *I*_in_ was converted to voltage *V*_out1_ when it flowed through a high value feedback resistor *R*1 (OHMITE model HVC4020V1008KET). Specifically, *R*1 is a 10 GΩ resistor with low tolerance (±10%) and a low temperature coefficient of resistance (±50 ppm/°C). During this conversion, an operational amplifier (amplifier 1) with femtoampere (1 fA) level input bias current *I*_b_ (RH < 50%, TA = 25 °C), low typical offset voltage drift (+0.13 μV/°C), and low (+8 μV) offset voltage (*V*_os_) were employed. The designed electrometer requires a ±5 V power supply. Therefore, the output voltage (*V*_out1_) can be calculated by the following equation:

(1) $$V_{\mathrm{out}1}=-I_{\mathrm{in}}R1+V_{\mathrm{os}}\left(1+\frac{R1}{Rs}\right)\pm I_{b}R1,$$

where *R*s is the internal resistance of the Faraday cup, and *R*s is typically great than *R*1. The output voltage (*V*_out1_) can then be simplified to

(2) $$V_{\mathrm{out}1}=-I_{\mathrm{in}}R1+V_{\mathrm{os}}\pm I_{b}R1,$$

A mica capacitor with ultra-low leakage current was also employed as the capacitance (*C*1). The response of the electrometer to time-varying signals could then be described by the rise time. Rise time of an analog output is generally defined as the time necessary for the output to rise from 10% to 90% of the final value when the input signal rises instantaneously from zero to a fixed value. According to previous work [20], the *RC* time constant of a first order system and rise time (*t*_r_) are related and can be described by the relationship

(3) $$t_{r}=2.2\cdot R1\cdot C1,$$

In this study, the rise time of the electrometer with a feedback resistance of 10 GΩ and capacitance of 47 pF was approximately one second.

Second, a low-noise instrumentation amplifier (amplifier 2) was used to convert the voltage (*V*_out1_) from the range of −5 V to +5 V to the range of 0 V to 5 V. During this conversion, a low-noise, precision voltage references (2.5 V) with extremely low, 0.5 ppm/°C typical temperature coefficients and excellent, ±0.02% initial accuracy was used. Moreover, two precision resistances (*R*_01_ and *R*_02_) with the same value were introduced. Therefore, the output voltage after amplifier 2(*V*_out2_) could be expressed as:

(4) $$V_{\mathrm{out}2}=\;\frac{R_{02}}{R_{01}+R_{02}}\left(-I_{\mathrm{in}}R1+V_{\mathrm{os}}\pm I_{b}R1\right)+2.5,$$

Finally, a 24-bit, no latency, differential delta-sigma (Δ-Σ) ADC with 5 ppm INL and 5 µV offset was introduced to convert the sensitive analog signal directly to an anti-interference digital signal. One of the advantages Δ-Σ ADCs offer over conventional ADCs is on-chip digital filtering. Additionally, the speed was programmed at 6.9 Hz in this study, leading to a large oversampling ratio, and the converter enters the ultralow noise mode (200 nV RMS) with simultaneous 50/60 Hz rejection. As shown, the voltage reference was also one of the differential input, so the output of the ADC *V*_out_ could be expressed as

(5) $$V_{\mathrm{out}2}=\frac{R_{02}}{R_{01}+R_{02}}\left(-I_{\mathrm{in}}R1+V_{\mathrm{os}}\pm I_{b}R1\right),$$

Therefore, the input current could be simplified to

(6) $$I_{\mathrm{in}}=-\frac{R_{01}+R_{02}}{R_{02}\cdot R1}V_{\mathrm{out}}+\left(\frac{V_{os}}{R1}\pm I_{b}\right),$$

Theoretically, the value of *V*_out_ +2.5 V, 0 V, −2.5 V are corresponded to the input current *I*_in_ value of −500 pA, 0 pA, +500 pA respectively, indicating an output voltage of 5 μV per −1 fA of the input signal current.

In order to eliminate the noise cause by the PCB board, three different strategies were employed: (I) As shown by the red dashed line in Figure 2, a guard circuit was used to reduce leakage current. The guard buffer used in the circuit was a unity-gain amplifier that created a low impedance replica of the input common-mode voltage. The input was surrounded by a guard ring that was connected to the output of a guard buffer instead of being connected to ground. (II) An appropriate cleaning process was applied to remove contamination in the PCB board. The effective insulation resistance of an electrometer circuit can be substantially degraded if the insulators are contaminated. High purity clean-room grade isopropyl alcohol (IPA) was used to clean the PCB board. Ultrasonic cleaners are also highly effective. The PCB board was then flushed with fresh IPA to remove any contaminants suspended in the solvent and then baked in an oven at elevated temperature to evaporate any residual moisture. (III) The PCB board was sealed in the electrometer circuit housing and pumped with high purity clean-room grade carbon dioxide (CO_2_) because the stability was observed to be highly sensitive to humidity variations [20]. A stabilized temperature was essential for proper functioning of the electrometer because the zero offsets are temperature dependent. As a result, a temperature control circuit was employed to ensure the temperature stability of the electrometer.

### 2.2. Calibration of the Aerosol Electrometer

**Figure 3:**
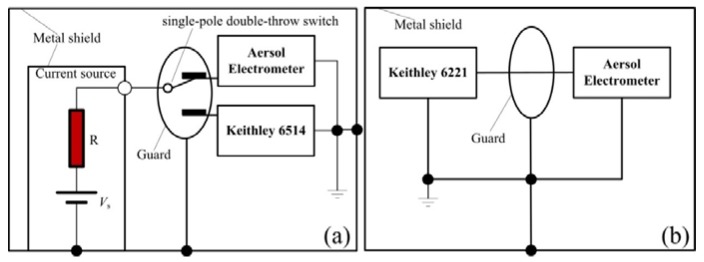
Schematics diagram of the experimental setup for aerosol electrometer calibration. (**a**) For the range of (0–10) fA; (**b**) for the range of (−500–+500) pA.

To calibration the aerosol electrometer, the experimental setup was built and the schematic diagram is shown in Figure 3. A high-impedance current source was applied to test the sensitivity at the range of 0 fA to 10 fA (Figure 3a). It consisted of a high standard resistor of 10 TΩ with a percent error of 10% (OHMITE, UM050E100BKE) and an adjustable voltage source in the range of 0 to 100 mV with a 1 mV resolution. According to Ohm’s law, the output current range was 0 fA to 10 fA. A commercial system electrometer (Keithley 6514, Solon, OH, UAS) was then applied to calculate the output current. The Keithley 6221 was also introduced to the calibration at the range of −500 pA to +500 pA because it is capable of high precision output and low current (resolution 100 fA @<2 nA). Moreover, the metal shield and guard were employed and connected to ground.

### 2.3. Experimental Setup for the Evaluation of the Aerosol Electrometer

**Figure 4:**
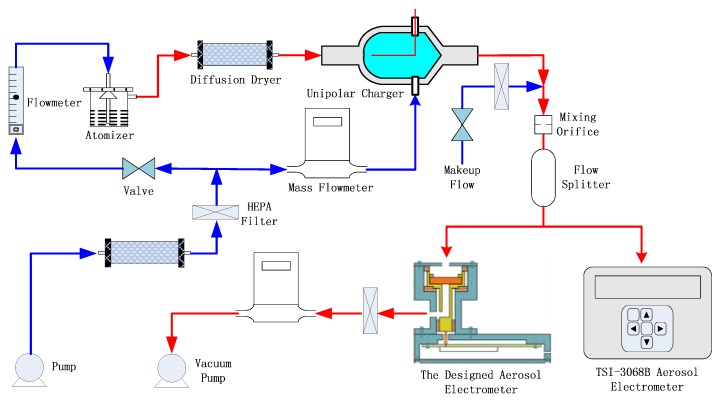
The schematics diagram of the experimental setup for evaluation of the aerosol electrometer.

The schematic diagram of the experimental setup for the evaluation of the aerosol electrometer is shown in Figure 4. It consisted of a clean air supply module, aerosol generator module, constant current charger module, a commercial aerosol electrometer (TSI-3068B, TSI, St. Paul, MN, USA) and the designed aerosol electrometer with a steady flow control module. In the clean air supply module, the clean air was generated when air was passed through the diffusion dryer and then a high-efficiency particulate-free air (HEPA) filter. The clean dry airflow was regulated and controlled by a mass flow meter and controller, typically in the range of 0 to 10 lpm. The clean air was divided into two parts with one part going to an atomizer and the other to a unipolar charger. The air flowing to the unipolar charger was set to a flow rate of 1 lpm by a mass flow meter and controller (Sevenstar CS200, NAURA Technology Group Co., Ltd., Beijing, China), and air flowing to the atomizer was set to a flow rate of 2 lpm by a valve. Sodium chloride (NaCl) particles were generated by a MetOne 255 atomizer (Met One Instruments, Inc., Medford, OR, USA). These particles were delivered to a diffusion dryer for water removing, and then to the unipolar charger to be charged. The constant current charger module was designed and evaluated previously [21]. A commercial aerosol electrometer (TSI-3068B) was used as a comparison to the designed aerosol electrometer and measured the charges on charged particles from 0.002 to 5 μm at the range of −12.5 PA to 12.5 PA. The flow rates for both electrometer were set to 2 lpm. In order to keep the particle diffusion losses the same, the two flow paths were symmetrical. This was accomplished by ensuring that the flow rates and conductive silicone tube lengths from the flow splitter to the designed aerosol electrometer/TSI-3068B inlet were the same.

## 3. Results

### 3.1. Calibration Results

#### 3.1.1. Linearity

**Figure 5:**
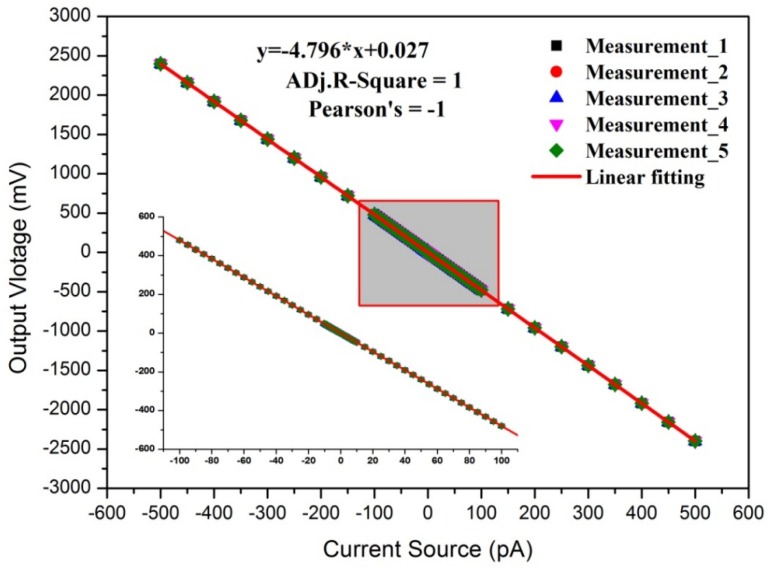
The calibration results of the aerosol electrometer and its linear fit.

**Figure 6:**
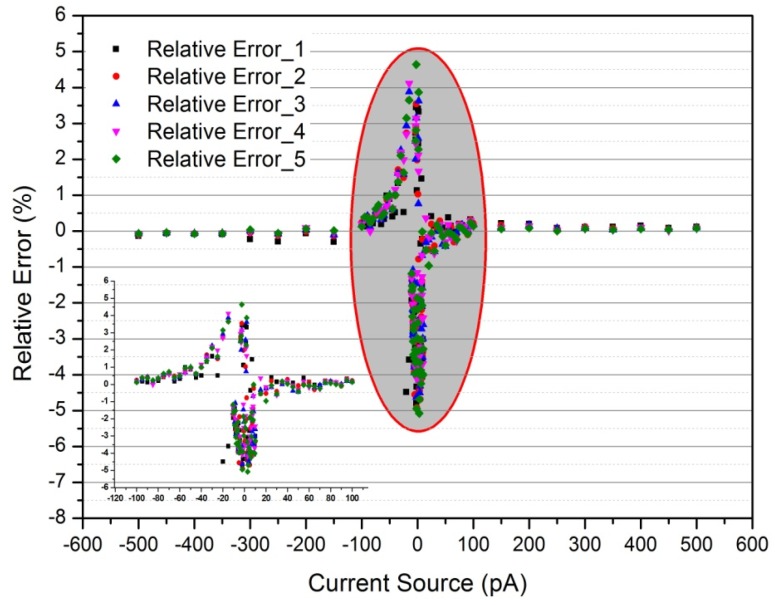
The relative error after linearity fitting.

Based on the above experimental setup shown in Figure 3b, linearity was tested over the range of −500 pA to +500 pA with a sampling time of 1 s. To confirm the stability and repeatability of the linearity of the designed aerosol electrometer, 5 replicate tests were performed. The linear fit is shown in Figure 5 and its relative error is shown in Figure 6.

As shown in Figure 5, the *x*-axis represents the input current from the Keithley 6221 and the *y*-axis represents the value of output voltage from the designed aerosol electrometer. The results of the 5 replicate tests exhibited and a good linear fit between the input current and output current. The detailed results at the range of −100 pA to 100 pA are also shown. The results showed great agreement with the theoretical equation described in Equation (5). Additionally, the Adj. R-Square and the Pearson’s correlation coefficient were both 1 further confirming the excellent fit and good linear relationship. When the fitting result was compared to Equation (5), the actual value of high value feedback resistor R1 was 9.5926 GΩ. The stability and repeatability of the aerosol electrometer were confirmed by the 5 replicate tests. As shown in Figure 6, the relative errors were controlled within 5.0%, 1.0% and 0.3% over the range of −40 pA to+40 pA, ±40 pA to ±100 pA, and ±100 pA to ±500 pA respectively.

#### 3.1.2. Sensitivity

**Figure 7:**
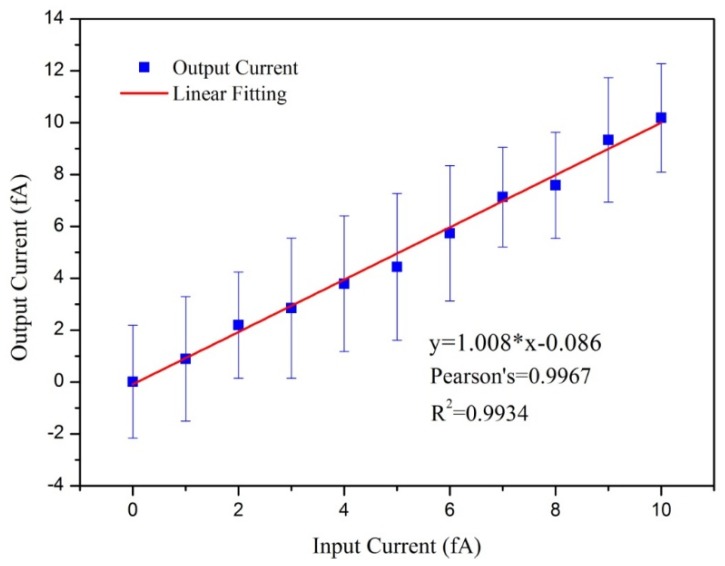
The results of sensitivity and its linear fit.

On the basis of the above experimental setup shown in Figure 3a, the sensitivity was tested at a sampling time of 1 s within the range of 0 to 10 fA and with interval of 1 fA. Using the above linear calibration, the results were calibrated and converted to current rather than voltage and the results are shown in Figure 7.

In Figure 7, the *x*-axis represents the input current and the *y*-axis represents the output current from the designed aerosol electrometer. The results and associated error bars showed an average peak-to-peak error of approximately 4.7 fA. A linear fit between the input current and output current was also performed. The Adj. R-Square and the Pearson’s correlation coefficient were both larger than 0.99 demonstrating an excellent fit and a good linear relationship. As a result, the sensitivity of the designed aerosol electrometer was determined to be approximately 1 fA.

### 3.2. Effect of Temperature on the Noise of the Aerosol Electrometer

Thermal energy produces motion of charged particles in any resistance. The charge movement results in noise often referred to as Johnson noise. Given that the electrometer is subject at least to the Johnson noise, it has been used as reference the limit noise. The Johnson current noise [20] can be stated as

(7) $$I_{RMS}=\;\frac{\sqrt{4kTBR}}{R}\;\mathrm{amperes},$$

where *R* is the source resistance in parallel with the input resistance of the electrometer, and *R* is typically equal to *R*1 = 9.5926 GΩ. *k* is Boltzmann’s constant (1.38 × 10^−23^ J/K), *T* is absolute temperature in *K*, and *B* is noise bandwidth in Hz, typically *B* = 1/(4*RC*). *C* is the sum of all capacitance shunting the input to the electrometer, including but not limited to the input capacitance and cable capacitance.

**Figure 8:**
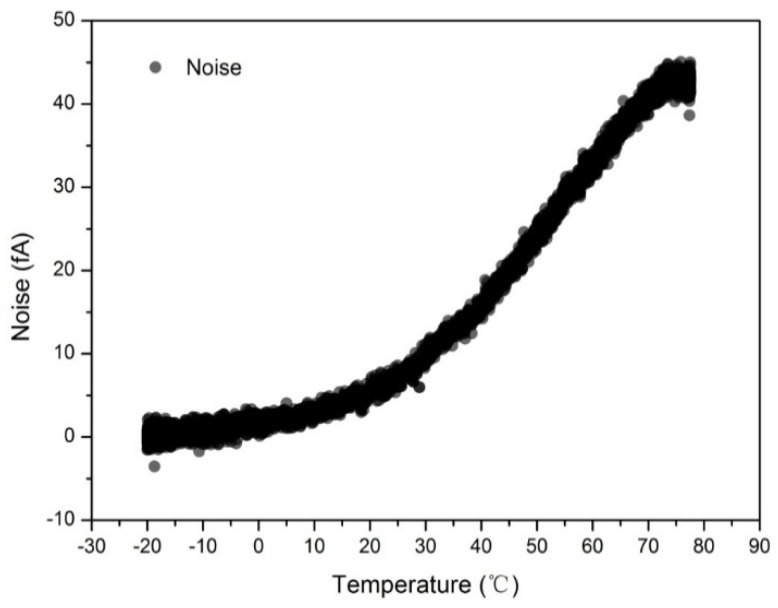
Effect of temperature on the noise of the electrometer.

The influences of temperature on electrometer have been aware for a long time [22]. It is commonly accepted that the performance of the aerosol electrometer were highly determined by the characteristics of electrometer [23]. In this study, two experiments were carried out to test the effect of temperature on the electrometer noise and the designed aerosol electrometer noise. In Experiment (I), the electrometer was placed in a commercial high-low temperature test chamber (Hongze, HKT705P-10) that operated from −25 °C to 80 °C with a 2 °C/min heating rate. However, the temperature variations to the electrometer exhibited a time lag when compared to the high-low temperature test chamber. As a result, a temperature sensor was installed inside the electrometer and the sensor recorded the entire temperature changing process from −20 °C to 75 °C. Corresponding to the actual temperature of the electrometer, noise was acquired when there was no input. Typical electrometer noise due to temperature variations is shown in Figure 8.

**Figure 9:**
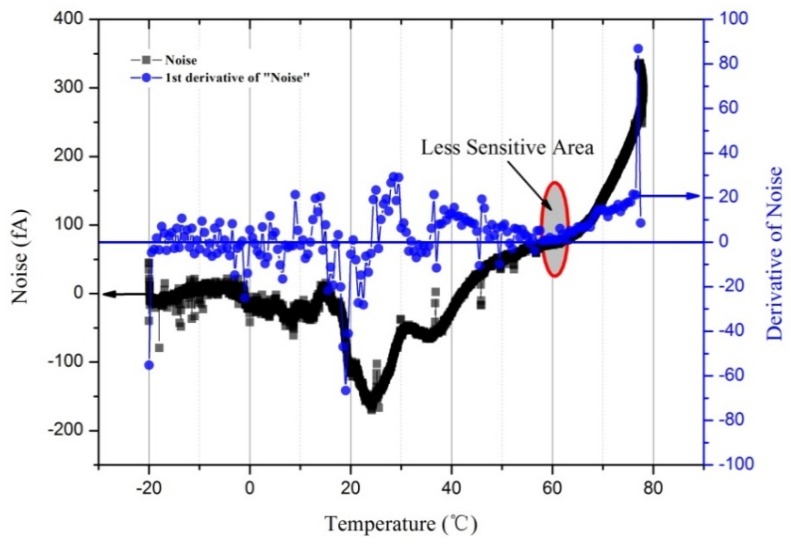
Effect of temperature on the noise of the aerosol electrometer.

In Experiment (II), the designed aerosol electrometer, with the same electrometer as was used in Experiment (I), was placed in the commercial high-low temperature test chamber. The experiment was run with the same experimental conditions as Experiment (I) except that there was no charged aerosol input in this experiment. A typical designed aerosol electrometer noise due to temperature variations is shown in Figure 9. The first derivative of noise with respect to temperature (interval of 0.5 °C) was also calculated.

**Figure 10:**
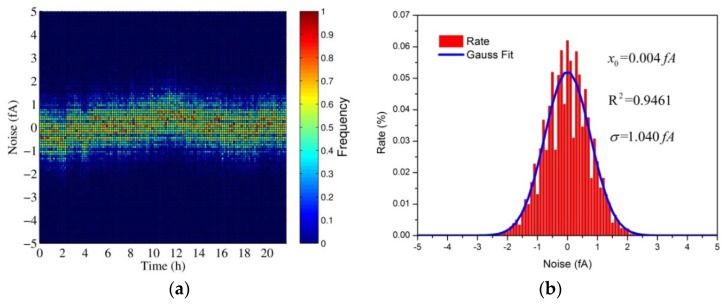
The noise of the aerosol electrometer after temperature control. (**a**) The scatter plot of noise; (**b**) the histogram of noise and its Gaussian fit.

As shown, the electrometer noise and the designed aerosol electrometer noise were both highly sensitive to temperature variations. In fact, the designed aerosol electrometer was more sensitive to temperature variations than the electrometer itself. This suggests that other components in the aerosol electrometer were also affected by temperature variations and generated current noise drift. Varying temperature can affect noise in several ways [20], including causing thermal expansion or contraction of insulators to produce noise currents, increasing the input bias current of the electrometer, and producing thermoelectric noise between various materials. To minimize errors due to temperature variations, it is essential to operate the entire system in a thermally stable environment rather than to only thermostatically treat the electrometer itself. Therefore, the designed aerosol electrometer should be controlled in a proper temperature. The results showed that the first derivative around the 60 °C ± 2 °C rounded to zero. This suggested that the noise in this area was less sensitive to temperature variations than in other areas. As a result, the temperature of the designed aerosol electrometer was controlled to be 60 °C ± 0.5 °C and then zeroed after achieving thermal stability. According to Equation (7), the theoretical limit of the current noise (*I*_RMS_) was calculated to be 1.031 fA. Statistical considerations show that peak-to-peak noise will be within five times of the root mean square (RMS) noise more than 99% of the time [20]. Therefore, the peak-to-peak noise (*I*_p-p_) was calculated to be 5.155 fA. To test the actual noise after temperature control, an experiment was carried out in which the noise was tested for more than 20 h. The results are shown in Figure 10.

As shown, the noise of the aerosol electrometer after temperature was controlled obeys a Gaussian distribution. The RMS noise (*I*_RMS_) with respect to the mean was 1.040 fA, and the peak-to-peak noise (*I*_p-p_) was 5.2 fA. In conclusion, the noise of the aerosol electrometer after temperature was controlled was very close to the theoretical limit of the current noise.

### 3.3. Results of the Evaluation of the Aerosol Electrometer

**Figure 11:**
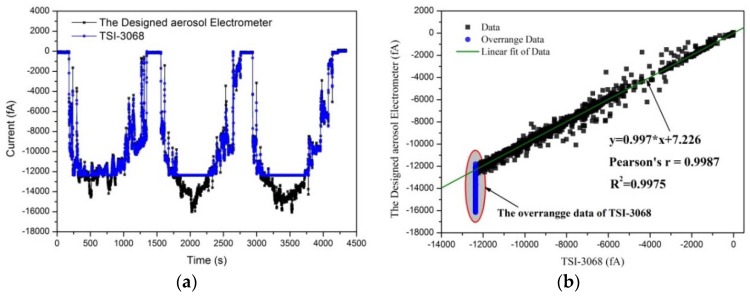
Results of the aerosol electrometer compared to the TSI-3068. (**a**) The measurement results; (**b**) the correlation.

Following the experimental setup shown in Figure 4, an experiment was carried out to evaluate the performance of the designed aerosol electrometer by comparing it to a commercial aerosol electrometer (TSI-3068B). The charged particle concentration was regulated by adjusting the voltage of the trap embedded in the unipolar charger. Figure 11a shows the measurement results for the set of 4500 s. As shown in Figure 11b, a 99.7% (R^2^) statistical correlation was obtained between the reading of the TSI-3068B and the reading of the designed aerosol electrometer, when they were used to simultaneously sample charged particles. The wide dynamic range of the designed aerosol electrometer, gave it an edge over the TSI-3068B because it operated well even when an overrange phenomenon appeared in the TSI-3068B.

## 4. Conclusions

In this study, a low-noise aerosol electrometer with wide dynamic range was designed to measure the total net charge on aerosol particles within the range −500 pA to +500 pA. The performance of the aerosol electrometer was evaluated by experiments that determined linearity, noise and sensitivity. The results showed that the relative errors were controlled within 5.0%, 1.0% and 0.3% over the range of −40 pA to+40 pA, ±40 pA to ±100 pA, and ±100 pA to ±500 pA respectively. To investigate the effect of temperature on the noise of the aerosol electrometer, two experiments were carried out on the electrometer and the aerosol electrometer. The results showed that the electrometer noise and the designed aerosol electrometer noise were both highly sensitive to temperature variations, and that the designed aerosol electrometer was more sensitive to temperature variations than the electrometer itself. This suggests that other components in the aerosol electrometer (aside from the electrometer itself) were also affected by temperature variations and generated current noise drift. Therefore, it was essential to operate the entire system in a thermally stable environment rather than only thermostatically treating the electrometer itself. The experimental results also showed that noise was less sensitive to temperature variations when the temperature was controlled to be 60 °C ± 2 °C. Based on theoretical analysis, the theoretical limit of the current noise (*I*_RMS_) was calculated to be 1.031 fA, and *I*_p-p_ was calculated to be 5.155 fA. After controlling the temperature, the experimental results showed that the RMS noise (*I*_RMS_) with respect to the mean was 1.04 fA and the peak-to-peak noise (*I*_p-p_) was 5.2 fA. This suggests that after the temperature was controlled, the aerosol electrometer noise was very close to the theoretical limit of the current noise. Finally, the performance of the designed aerosol electrometer was further evaluated by comparing it to a commercial aerosol electrometer (TSI-3068B). The excellent correlation and the advantage of wide dynamic range were demonstrated. A 99.7% (R^2^) statistical correlation was obtained between the TSI-3068B reading and the designed aerosol electrometer reading when they were used simultaneously to sample charged particles.
